# Temporal changes in chronic disease management in primary care in relation to telehealth policy changes: Australian whole-of-population interrupted time-series analysis

**DOI:** 10.1093/fampra/cmag042

**Published:** 2026-06-25

**Authors:** Danielle C Butler, Nina Lazarevic, Grace Joshy, Christine Phillips, Sally Hall Dykgraaf, Jennifer Welsh, Kirsty A Douglas, Hsei Di Law, Emily Banks, Jane Desborough, Tsheten Tsheten, Jason Agostino, Susan Trevenar, Rosemary J Korda

**Affiliations:** National Centre for Epidemiology and Population Health, The Australian National University, Acton, Australian Capital Territory 2601, Australia; National Centre for Epidemiology and Population Health, The Australian National University, Acton, Australian Capital Territory 2601, Australia; National Centre for Epidemiology and Population Health, The Australian National University, Acton, Australian Capital Territory 2601, Australia; School of Medicine and Psychology, Australian National University, Australian Capital Territory 2601, Australia; School of Medicine and Psychology, Australian National University, Australian Capital Territory 2601, Australia; National Centre for Epidemiology and Population Health, The Australian National University, Acton, Australian Capital Territory 2601, Australia; School of Medicine and Psychology, Australian National University, Australian Capital Territory 2601, Australia; National Centre for Epidemiology and Population Health, The Australian National University, Acton, Australian Capital Territory 2601, Australia; National Centre for Epidemiology and Population Health, The Australian National University, Acton, Australian Capital Territory 2601, Australia; National Centre for Epidemiology and Population Health, The Australian National University, Acton, Australian Capital Territory 2601, Australia; National Centre for Epidemiology and Population Health, The Australian National University, Acton, Australian Capital Territory 2601, Australia; National Centre for Epidemiology and Population Health, The Australian National University, Acton, Australian Capital Territory 2601, Australia; National Centre for Epidemiology and Population Health, The Australian National University, Acton, Australian Capital Territory 2601, Australia; National Centre for Epidemiology and Population Health, The Australian National University, Acton, Australian Capital Territory 2601, Australia

**Keywords:** telehealth, general practice, health policy, linked data, chronic disease management

## Abstract

**Background:**

Universal telehealth aims to support access to timely coordinated chronic disease care. Large-scale evidence on the extent to which this occurs to guide telehealth policy is limited.

**Objective:**

To examine temporal changes in uptake and timeliness of general practitioner chronic disease management (GP-CDM) services in Australia following universal telehealth introduction (March 2020) and removal of subsidized telephone (but not video) GP-CDM services (July 2021).

**Methods:**

Whole-of-population cohort study of linked national claims and death data, 2018–2022. Interrupted time-series analyses quantified temporal changes in GP-CDM service uptake and timeliness following telehealth policy changes.

**Results:**

From 2018 to 2022, each month an average of 568 858 GP-CDM services were delivered, with 25–43 users and 44–76 services per 1000 population aged 45–<85 per month. After universal telehealth introduction, GP-CDM uptake remained stable, with similar trends pre- and early-pandemic. Monthly uptake decreased substantially following the removal of telephone GP-CDM services [decrease of 4.0 users (95%CI −6.3, −1.7) and 6.9 services (−10.5, −3.3) per 1000 population]. In the first month of telehealth, 38.7% of people using GP-CDM services used telehealth (37% telephone, 1.7% video), declining to 2.2–5.4%/month after removal of telephone GP-CDM services. Small improvements in GP-CDM timeliness stalled once telephone services were no longer available. Patterns were similar across population subgroups.

**Conclusions:**

Telehealth policies in Australia sustained access to chronic disease care during the pandemic. Limiting access to these services to video alone was associated with a greater than expected decline in use had the pandemic and introduction of telehealth not occurred.

Key messages

- Large-scale evidence is lacking on telehealth policies and chronic disease care.
- Universal telehealth introduced in Australia supported GP chronic disease care.
- Subsequent policies removed access to chronic disease services by telephone.
- Uptake then declined to lower than pre-pandemic and telehealth introduction.

## Introduction

Chronic disease is the key driver of premature mortality, disability and healthcare costs, accounting for over two-thirds of the global burden of disease (1). High-quality primary care plays a critical role in improving chronic disease outcomes—including reduced mortality rates (2, 3), improved medication adherence (4) and quality of life (5), and fewer emergency department visits and hospital admissions (2, 3)—through timely, comprehensive and coordinated multidisciplinary team-based care, centred on patient-identified goals (6). In many countries, these services were offered by telehealth for the first time in 2020, which was critical in the recovery and maintenance of chronic disease management (CDM) services following initial disruptions in the COVID-19 pandemic period (7–13). Telehealth policies have since evolved, including more restricted access to telehealth in some jurisdictions (14); however, ongoing access to telehealth may be important for maintaining access to CDM services. To our knowledge, no studies have quantitatively examined ongoing telehealth policy changes in relation to the uptake and timeliness of chronic disease care, particularly beyond the early pandemic period.

In Australia, where one in two adults has a chronic disease (15), chronic disease care activities are subsidized by Medicare, Australia's universal health insurance scheme, through the CDM program (16). Under this program, a range of subsidized services are available for general practitioners (GPs, Australia's primary care physicians) to undertake chronic disease and complex care planning and management in collaboration with patients and other health professionals involved in their care (see box). More than 3.8 million Australians had a CDM service in 2019 (∼8% of all GP services), at a cost of $1 billion (17). Evidence indicates that receipt of these services improves monitoring of chronic disease (18), medication adherence, and reduces hospitalizations (19) and mortality (20). Chronic Disease Management services were only available as in-person consultations until equivalent telehealth services by telephone and video were introduced in March of 2020, as part of Australia's COVID-19 pandemic response to ensure access to primary care through universal telehealth.

Consistent with the growth in the population living with chronic disease globally, uptake of GP-CDM services in Australia increased over time prior to the pandemic (17). After a decrease in March and April of 2020, the total number of GP-CDM services over the remainder of the first year of the pandemic was comparable to or higher than the preceding year (8, 17). Around a quarter of these services were delivered by telehealth, with > 95% by telephone, i.e. audio only (17). In July 2021, subsidized telephone services for GP-CDM services were removed from the Medicare schedule on the assumption that, where telehealth was required, video was the optimal modality for complex consultations, including CDM (21). While changes in levels of GP-CDM services have been described for the first year of the pandemic (17), there has been no analysis of temporal patterns to assess the relation of telehealth policies with uptake of GP-CDM services, including post-2020.

The objective of this study was to use whole-of-population linked data to examine temporal changes in the uptake of GP-CDM services in relation to key telehealth policy changes, including by key sociodemographic characteristics. We use the Australian Medicare-funded CDM program as our focus. Specifically, we quantify and analyse trend changes in uptake and timeliness of GP-CDM services—including among new and pre-existing users—over the first 3 years of the pandemic and in relation to the introduction of telehealth CDM items (pre-post April 2020) and subsequent policy changes to remove subsidized telephone (but not video) GP-CDM services (July 2021).

## Methods

### Data sources

We used data from the Person Level Integrated Data Asset (PLIDA), a secure data asset combining information on health, education, government payments, income and taxation, and population demographics (including the Census). Underpinning PLIDA data is the Person Linkage Spine (hereafter called the Spine), a person-level identification key created by linking data from key administrative databases, including the Medicare Consumer Directory (records of those enrolled in Medicare, resulting in virtually complete coverage of the resident Australian population (22). For this study, we used linked Medicare Consumer Directory, Medicare Benefits Schedule (MBS) claims (1 January 2016–31 December 2022), migration (to December 2022) and Death Registrations (to 2021) data. Data were linked indirectly via the Spine (23) using deterministic linkage methods based on name, full date of birth, address and sex. A direct link exists between Medicare enrolments, MBS data and the Spine.

The Medicare Benefits Schedule data contain information relating to claims for medical services that are reimbursed under Medicare, including visits to GPs and other doctors outside a hospital (identified by specific MBS item numbers). All Australian citizens, permanent residents and those from countries with reciprocal arrangements in place are eligible for Medicare subsidised services. Death Registrations data contain the date of death for all deaths registered in Australia, and for this project, were available for the 2016 to 2021 calendar years (24).

### Study population

The study population was defined on a rolling monthly basis for the period 2018–22. For each month, individuals aged 45–<85 years, enrolled in Medicare, residing in Australia, and who received at least one MBS service in the last 2 years were included. Given we were unable to identify people with a chronic condition, and hence eligible for a chronic disease service, we limited our study population to those aged 45–<85 years and most likely to be affected by a chronic disease (70% Australian adults aged over 45 years (25)). We excluded those who were absent from Australia or died anytime during that month or prior.

### Variables

While much of CDM in primary care occurs during routine consultations, the use of specific GP-CDM services provides an indication of overall trends in CDM and coordination in the Australian population. CDM services can only be claimed for patients with one or more chronic medical conditions. See Summary Box for details.

Summary Box: Chronic Disease Management (CDM) program and peri-pandemic policy changes in Australia. ***Services available under the CDM Program***

“A **General Practitioner Management Plan (GPMP),** which is a plan of action agreed between a patient and their GP. The plan identifies the patient’s health and care needs, sets out the services to be provided by the GP, and lists the actions the patient can take to help manage their condition.

**“Team Care Arrangements (TCAs),** for patients with complex care needs requiring multidisciplinary care, which provide Medicare-subsidised care (5 services per calendar year) from selected allied health care providers for individual treatment services where the patient also has a GPMP.”(21)

**Review of GPMPs and TCAs**, to support regular review of plans by the GP and patient. A review involves checking that a patient’s goals are being met through the plan and provides an opportunity to make any adjustments needed.

**Monitoring and support services** for a person with a chronic disease provided by a practice nurse or Aboriginal and Torres Strait Islander health practitioner to review clinical and self-management progress.

**Individual and group allied health services** where care is deemed beneficial to their condition by their GP. A maximum of 5 individual and 8 group services (any type) may claimed per calendar year.

***Relevant policies introduced during the peri-pandemic period***

**March-April 2020,** Medicare subsided universal telehealth (telephone and video services) introduced, including for CDM services

**July 2021**, subsided telephone GP CDM services ceased; subsided video GP-CDM services and telephone and video services for monitoring and support and allied health services continued.

To examine temporal changes in uptake of GP-CDM services, our study outcomes were: (i) proportion of the study population using GP-CDM services [general practitioner management plan (GPMP) or team care arrangement (TCA) or GPMP/TCA review MBS service (review)] in each month, overall and by modality (i.e. in-person, by video or by telephone); (ii) rates of GP-CDM services among the study population in each month, overall and by modality. In Australia, there are no additional incentives or formal support to communicate with care providers by video (e.g. technical support or subsidies for equipment such as video cameras or data plans).

To examine trend changes in timeliness of GP-CDM services, our study outcomes were (iii) overdue for a GP-CDM service: proportion of the study population who had a GPMP/TCA in the last 2 years (pre-existing users), who did not have a GPMP/TCA or review in the last 12 months; (iv) average number of days to review since the last GP-CDM service for pre-existing users who had a review in that month and who had a previously recorded GP-CDM service.

For patient characteristics, we obtained sociodemographic information from the Medicare Consumer Directory, including age (based on date of birth), sex, remoteness area, and socioeconomic status (measured with Socio-Economic Indexes for Areas and Index of Relative Socioeconomic Advantage and Disadvantage (26), derived from Statistical Area Level 1), and state or territory.

### Analysis

First, we describe trends over time in uptake of CDM among the study population between 1 January 2018 and 31 December 2022, in total and separately by modality and by sociodemographic characteristics. As the 2022 death registrations were not available to researchers at the time of the study, we weighted the time-series denominators in 2022 using age- and sex-specific estimates of the cumulative probability of survival in each month based on death registrations data 2018–2021.

To examine outcomes over time, and specifically in relation to policy initiatives, we conducted interrupted time-series analysis, using dynamic regression models with autoregressive integrated moving average (ARIMA) errors, which take into account autocorrelation and seasonality (27, 28). We estimated the impact of each policy change—(i) The start of the pandemic and introduction of universal telehealth 1 April 2020, and (ii) the removed of telephone GP-CDM services in 1 July 2021—using transfer functions, which allow complex relationships between the interruption and the outcomes (e.g. an instantaneous increase in an outcome followed by a gradual return to baseline over time). The shapes of these functions and the modelling procedure are detailed in the Supplemental Material. We used these models to estimate what the trends in use of CDM would have been had the policies to introduce universal telehealth and subsequently remove subsidized telephone services not been implemented (i.e. the counterfactual time series, calculated as the fitted time series minus the modelled interruption impact). To account for the potential confounding by public health restrictions in relation to COVID-19 outbreaks, we controlled for the number of new COVID-19 cases in each month, obtained from the Australian National Notifiable Diseases Surveillance System data collection (29), which we modelled using a transfer function (27, 28). We also examined time series separately by key sociodemographic characteristics to determine whether trends over time varied across population subgroups. In sensitivity analysis, we examined time series including people aged 85–99.

In supplementary time-series analysis, to determine if policy initiatives may have been associated with changes in uptake by patients using CDM services for the first time, we also examined the proportion of the study population using a GPMP/TCA service for the first time in the last 2 years (new users), overall and by modality. For this outcome, we restricted the population denominator to people who had no claim for GP-CDM services in the last 2 years (i.e. non-users). We further examined monthly rates of CDM monitoring/support and allied health services provided to pre-existing users separately for monitoring and support (provided by a practice nurse or Aboriginal and Torres Strait Islander health practitioner) and allied health services, including by modality and by state or territory.

Stata 17 and R v4.4.0 (TSA (28) and forecast (30) packages) were used for the analysis, completed in DataLab, a secure remote access computer facility for analysis of data compiled and managed by the Australian Bureau of Statistics. We obtained ethics approval for this study from The Australian National University Human Research Ethics Committee (HREC 2021/619).

## Results

### Study population characteristics

For each month from January 2018 to December 2022, the total number of adults aged 45–< 85 years who met our inclusion criteria ranged from 8 995 850 to 9 947 124 (mean 9 520 537). The number of GP-CDM services ranged from 412 760 to 739 067 (mean 568 858) (Supplementary Table S1).

### Changes over time in use of GP-CDM services

**Figure 1:**
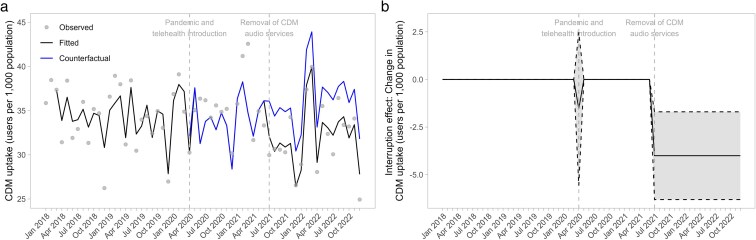
GP-CDM uptake per 1000 population aged 45–<85, by month, January 2018–December 2022. Panel (a) shows the observed time series (grey points), fitted time series (black line) and estimated counterfactual series in the absence of interruptions (blue solid line), controlling for COVID-19 cases. Panel (b) shows the estimated change associated with the interruptions, with 95% confidence intervals. Note. Denominator is the population in each month who were aged 45–<85 years, enrolled in Medicare, alive, residing in one of the eight major states or territories, and not physically absent from Australia. In panel (a), the counterfactual and fitted lines are equal between May 2020 and June 2021 as the estimated interruption effect was zero. Given the estimated effect from May 2020 to June 2021 was zero, the fitted and counterfactual lines are on top of each other in panel (a).

Uptake—GP-CDM users and services: From 2018 to 2022, the proportion of the study population using CDM services in each month ranged from 25 to 43 users and 44 to 76 services per 1000 population aged 45–<85 (Fig. 1a;  Supplementary Table S1). Following universal telehealth introduction at the start of the pandemic, after a brief non-statistically significant drop [1.5 users (95%CI −5.6, 2.5) per 1000 population in April 2020], uptake of GP-CDM services returned to stable pre-pandemic levels between May 2020 and June 2021 (30–43 users per 1000 population each month) (Fig. 1a and b). After removal of telephone CDM services in July 2021, there was an immediate but sustained decrease in uptake from July 2021 [decrease of 4.0 users (95%CI −6.3, −1.7) per 1000 population] (Fig. 1a and b). This corresponds to a decline in the number of users of 39 327 in July 2021.

Patterns in uptake for new users were similar to those observed for overall use in that there was an immediate decrease in new users after the removal of telephone CDM services [2.1 (95%CI −3.1, −1.1) fewer new users per 1000 population, 13 049 fewer new users in July 2021] (Supplementary Figure S1; Supplementary Table S1). However, there was a gradual return to pre-telehealth/pre-pandemic levels of new users towards the end of the study period.

**Figure 2:**
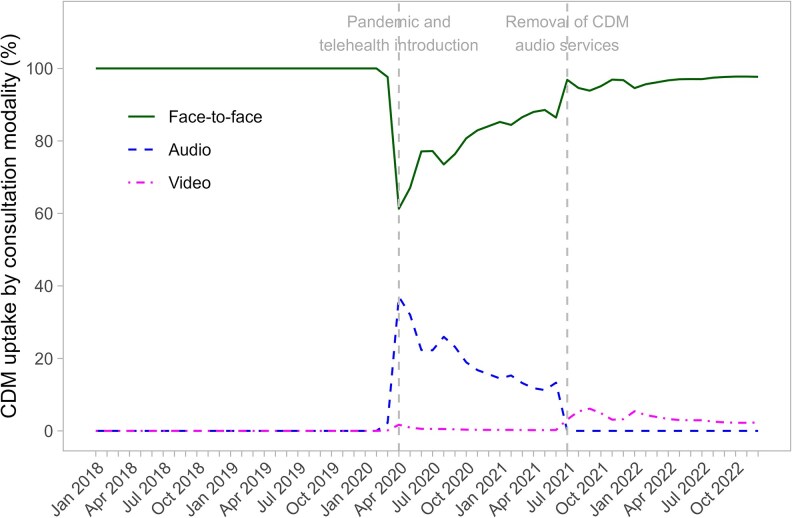
GP-CDM uptake, by month and modality. Note. Denominator is users of GPMP/TCA services among the eligible population (refer to note below Fig. 1).

In the first month of telehealth, April 2020, 38.7% of people who used a GPMP or TCA received these services by telehealth (37.0% telephone, 1.7% video), with the proportion gradually declining thereafter to 13.6% in June 2021 when reimbursement for telephone GPMP/TCA items ceased (Fig. 2). There was minimal replacement by video thereafter (ranging from 2.2% to 5.4% per month in 2022).

**Figure 3:**
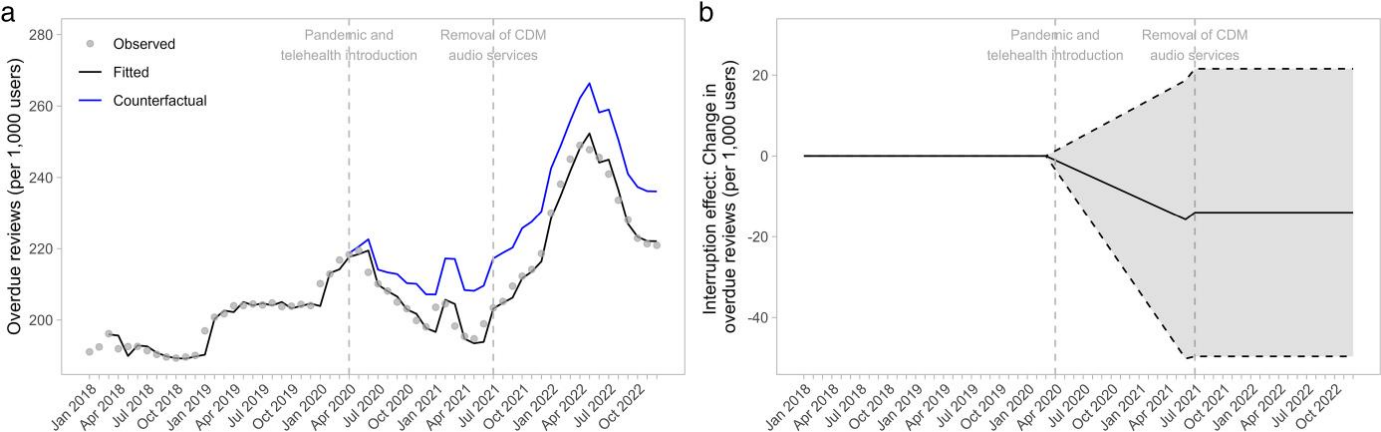
CDM users who were overdue for a review per 1000 pre-existing users aged 45–<85, by month, January 2018–December 2022. Panel (a) shows the observed time series (grey points), fitted time series (black line) and estimated counterfactual series in the absence of interruptions (blue solid line), controlling for COVID-19 cases. Panel (b) shows the estimated change associated with the interruptions, with 95% confidence intervals. Note. Denominator is the population in each month who were aged 45–<85 years, enrolled in Medicare, alive, residing in one of the eight major states or territories, not physically absent from Australia, and who had a claim for a GPMP/TCA in the previous 2 years.

**Figure 4:**
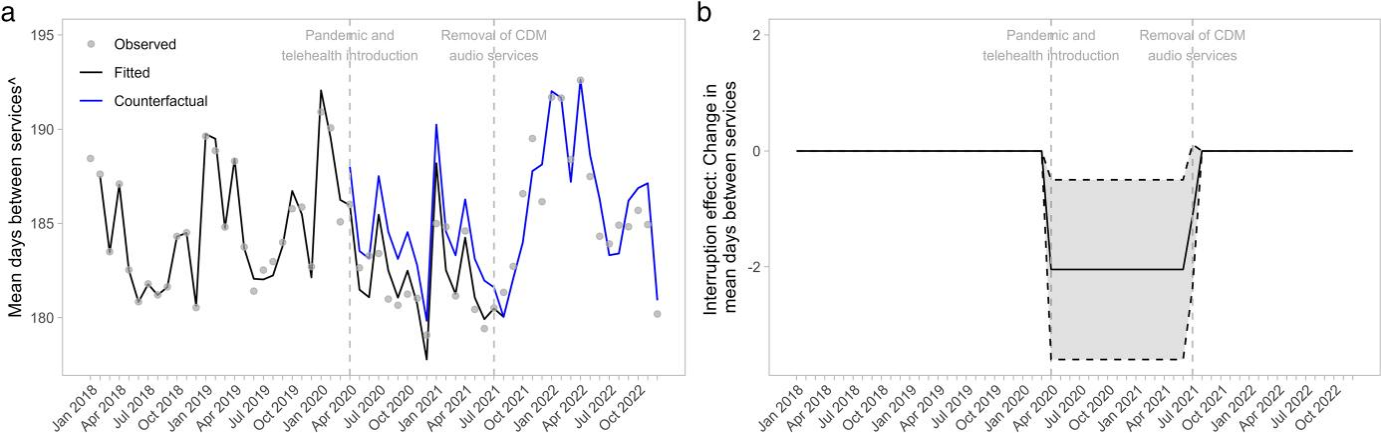
Mean number of days between GP-CDM services, by month, January 2018–December 2022. Panel (a) shows the observed time series (grey points), fitted time series (black line) and estimated counterfactual series in the absence of interruptions (blue solid line), controlling for COVID-19 cases. Panel (b) shows the estimated change associated with the interruptions, with 95% confidence intervals. Note. Denominator is the population in each month who were aged 45–<85 years, enrolled in Medicare, alive, residing in one of the eight major states or territories, not physically absent from Australia, and who had a claim for a GPMP/TCA in the previous two years. ^Denominator is restricted to those who had a review in the current month and at least one previous review since 2016, and time between services is capped at 2 years.

Timeliness—overdue users of GP-CDM services and time since last GP-CDM review: The proportion of current users overdue for CDM review increased over time until universal telehealth introduction and the start of the pandemic (peaking at 219 per 1000 current users who were overdue for review) (Fig. 3a; Supplementary Table S1). For those who had a review in that month, the average number of days since their last GP-CDM service between 2018 and 2022 ranged from 179 to 193 days (Fig. 4a; Supplementary Table S1). Following telehealth introduction and the start of the pandemic, there were small improvements in trends, with either substantial uncertainty in the estimated associations (overdue users) (Fig. 3b) or improvements not sustained once GP-CDM services were no longer available by telephone (days between services) (Fig. 4b).

Time series by sociodemographic characteristics: Generally, patterns were similar across age groups, area socioeconomic disadvantage, and remoteness (Supplementary Figures S2–S5).

### Changes over time in use of CDM monitoring/support and allied health services

During January 2018 to December 2022, the number of CDM monitoring/support services ranged from 32 to 98 per 1000 pre-existing users each month, and the number of CDM allied health services ranged from 99 to 183 per 1000 pre-existing users each month (Supplementary Figure S6; Supplementary Table S1). Patterns in uptake of CDM monitoring/support services and CDM allied health services were similar to those observed for GP-CDM services: rates were largely unchanged from those observed prior to the introduction of universal telehealth at the start of the pandemic, with a sustained decrease in both monitoring/support services and allied health services following the removal of telephone CDM services (Supplementary Figure S6a–d). Following the introduction of universal telehealth, the proportion of people using telehealth for CDM monitoring/support (telephone 2.9%–13.8% per month, April 2020–June 2021, video 0.2%–1.1%) and allied health services (telephone 0.4%–3.9%, video 0.1%–1.4%) was lower than for GP-CDM services (Supplementary Figure S7). Temporal patterns were similar across separate states and territories (Supplementary Figure S8).

### Sensitivity analyses

Patterns over time in outcomes were negligibly changed when including people aged 85–99 (Supplementary Figure S9).

## Discussion

### Summary of the principal findings

Following the introduction of universal telehealth, GP-CDM uptake during the pandemic was stable and similar to pre-pandemic uptake. This is likely due to the availability of telephone telehealth services. Subsequent policies to remove access to telephone-only consultations were associated with decreased uptake, with a decline in total and new users to lower than expected levels had the pandemic and introduction of telehealth not occurred (by 4.0 and 2.1 users per 1000 population, respectively; e.g. 39 327 fewer total users and 13 049 fewer new users in July 2021). With respect to the timeliness of GP-CDM services, initial small reductions in days between GP-CDM services were not sustained once these were no longer available by telephone.

### Comparison with pre-existing literature

To our knowledge, no studies have quantified changes in delivery or uptake of chronic disease care associated with the introduction of telehealth policy, or changes in these policies over time—nationally or internationally. Our study indicates that initial declines in use of GP-CDM services reported nationally in Australia early in the pandemic (8) were short-lived, not only for any use but also among new users, once long-term trends and seasonality are taken into account. Overall use of GP services demonstrated similar patterns (31). In fact, after the brief non-significant disruption, introduction of telehealth was not associated with a significant or persistent change in uptake, with trends indicating stability in line with long-term trends and seasonality. Internationally, marked disruptions to chronic disease care, including monitoring of and screening for chronic conditions, were reported (9–13, 32), with the subsequent recovery of these services partly attributed to the introduction of telehealth (7–9, 32). That similar longer-term disruptions in GP-CDM services were not observed in Australia may reflect differences in local telehealth policy settings, differences in background rates of COVID-19, and/or the fact that underlying long-term trends and seasonality were not accounted for in the other studies. Moreover, it appears that initial telehealth policy settings in Australia at least maintained access to chronic disease care during the pandemic. Further, changes in trends over time and in relation to telehealth policies were similar across age, remoteness and socioeconomic status, in contrast to international findings of racial and socioeconomic inequalities in initial disruptions to care and recovery of service levels with the pandemic (10, 12).

When available, virtually all GP-CDM telehealth services were by telephone rather than video, and removing access by telephone corresponded with a decline in use of GP-CDM services. This finding is consistent with qualitative studies, which have found that most people living with chronic disease preferred telephone over video consultations (7), and is reflected in the higher uptake of GP telehealth services by telephone rather than video overall (33). Qualitative data from Australia early in the pandemic indicated a disruption to CDM allied health services (7), with views expressed that these services could not be provided unless in person. Our findings suggest that this initial disruption was brief. Furthermore, similar to the uptake of GP-CDM services, services for monitoring/support and allied health CDM services were generally maintained until the removal of GP-CDM telephone services. Following this, there was a small but sustained decrease in CDM allied health services despite these still being available by telephone, video and in person. Eligibility for these services requires a TCA prepared by a GP; hence, this finding may be a downstream consequence of lower uptake of GP-CDM services observed following removal of access to these by telephone.

### Strengths and limitations

We used whole-of-population MBS data that includes all claims for medical services that are reimbursed under Medicare for all Australian citizens and permanent residents, linked to death registrations and migration data, with high linkage rates of 98% and 68%, respectively. There are important aspects of CDM in primary care not captured by these data. We did not use a set of chronic diseases in defining the outcome of being overdue, as we were unable to identify people with a specific chronic condition. Routinely collected primary care medical record data may shed some light on this; however, such data are not currently available for the whole Australian population for research purposes. The reductions in uptake of CDM services observed may reflect that CDM services by telephone were substituted for other available services, such as standard telephone or in-person services. The extent to which this occurred is unclear and cannot be assessed with these data because the reason for the visit is not available in the data. However, this is unlikely given that CDM services are remunerated by Medicare at a high rate relative to most other GP services (i.e. substitution would occur at a loss for practices). Furthermore, as noted above, eligibility for subsidized CDM allied health services requires a claim for a GP-CDM service. As such, it is likely that our findings reflect loss of these services and a reduction in best practice chronic disease care. Furthermore, while we have examined timeliness as an element of quality in addition to uptake, the actual quality of the services delivered cannot be determined; qualitative and ethnographic studies would be informative for this. Similarly, such studies would be informative for why uptake among new users only returned to baseline levels.

Our analyses used ARIMA dynamic regression models that account for autocorrelation and seasonality and allow control for confounders that have dynamic relationships with the outcome. We modelled outcomes in relation to policy changes using a range of transfer functions to allow for possible non-linear impacts that reverberate over time. Although we controlled for COVID-19 cases as a proxy for pandemic effects on primary care services, we did not have access to a related control outcome time series that was not affected by the pandemic and telehealth-related policy changes. Finally, changes over time may partly reflect GP availability; however, this was relatively stable over the study period (34).

### Implications for practice and future research

Team-based care planning and coordination have been shown to improve chronic disease morbidity and quality of life (3, 6), and as such, increased access has the potential to improve the health of the population. Using uptake of CDM services as an indicator of this type of care, our findings suggest that enabling access via telephone may improve uptake and ongoing use of these services. We found that while telephone services sustained use of these services in the early stage of the pandemic, the policy change to limit access to GP-CDM services via telehealth to video consultations only was associated with a decline in use to lower than pre-pandemic levels; such that there were an estimated 40 000 fewer users than would have been expected had the pandemic and telehealth introduction not occurred. As countries seek to maximize the benefits associated with team-based care for management of chronic disease, our findings highlight the importance of initiatives that support chronic disease care planning and coordination by telephone, in addition to by video or in-person.

Available evidence suggests that the telephone is similar in safety and effectiveness for managing continuing stable conditions (35). This is predicated on a pre-existing relationship between the healthcare provider and patient, no requirement for physical examination, and when occurring as part of a chain of in-person and telephone consultations (36, 37). Further research is needed to better quantify the relative safety and effectiveness of differing modalities for providing CDM in primary care. Working with policy-makers, this program of research has informed recommendations being considered by the Health Minister (37) to revise telehealth policy settings to better address known risks related to telehealth consultations. This is all the more pressing given our finding that the availability of CDM services via telephone supported access to these services. Recently, Australia has introduced reforms to support voluntary patient registration (38), similar to patient enrolment used in many other countries (39), which provides a mechanism for a range of policies to support continuity and multidisciplinary care, particularly for those with ongoing and complex health conditions. Similar to making longer standard telephone consultations available to those enrolled, GP-CDM services by telephone might also be made available, with the known caveats from current evidence. Finally, little is known about consumer perspectives on the use of telehealth for CDM, particularly now that this is routinely available across most jurisdictions internationally.

## Conclusion

The introduction of telehealth in Australia was associated with maintained access to chronic disease care during the pandemic. However, policies to limit access to these services via video alone were associated with a decline in use. Clarifying safety risks and implications for the quality of telehealth consultations—either by video or telephone—for CDM in primary care, and balancing this against access, is critical to inform and guide consumers and clinicians in integrating this modality in care.

## Supplementary Material

cmag042_Supplementary_Data

## Data Availability

All data for this project were accessed through the Person Level Integrated Data Asset (PLIDA). The Australian Bureau of Statistics is trusted as the accredited Integrating Authority for PLIDA and provides access to authorized researchers (40).
