# Supplementary material for: Temporal changes in chronic disease management in primary care in relation to telehealth policy changes: Australian whole-of-population interrupted time-series analysis
Source: Fam Pract. 2026 Jun 25;43(4):cmag042. doi: 10.1093/fampra/cmag042 (PMC13296993; doi:10.1093/fampra/cmag042)
Supplement: cmag042_Supplementary_Data [file cmag042_supplementary_data.pdf]

SUPPLEMENTARY MATERIAL: Temporal changes in chronic disease management in primary care in relation to telehealth policy changes: Australian whole-of-population interrupted time-series analysis

Authors

Danielle C Butler[1], Fellow, FRACGP, PhD, ORCID: 0000-0003-4870-4544 (Joint first author)
Nina Lazarevic[1], Research Fellow, PhD, ORCID: 0000-0001-6463-6719 (Joint first author)
Grace Joshy[1], Associate Professor, PhD, ORICD: 0000-0002-0718-6368
Christine Phillips[2], Professor, FRACGP, MD, ORCID: 0000-0001-5602-3664
Sally Hall Dykgraaf[2], Professor, RN, PhD, ORCID: 0000-0002-8532-1086
Jennifer Welsh[1], Fellow, PhD, ORCID: 0000-0003-4415-5920
Kirsty A Douglas[2], Professor, FRACGP, MD ORCID: 0000-0003-0174-782X
Hsei Di Law[1], Research Fellow, MSc, MPH, ORCID: 0000-0003-1590-0370
Emily Banks[1], Professor, PhD FAFPHM, ORCID: 0000-0002-4617-1302
Jane Desborough[1], Associate Professor, RN, PhD, ORCID: 0000-0003-1406-4593
Tsheten Tsheten[1], Research Fellow, PhD, ORCID: 0000-0002-8071-5721
Jason Agostino[1], Honorary Clinical Associate Professor, FRACGP, MPhil(AppEpi), ORCID: 0000-0001-7937-6526
Susan Trevenar[1], Senior Research Officer, BSc Hons (Psych), ORCID 0000 0002 1340 2725
Rosemary J. Korda[1], Professor, PhD, ORCID ID: 0000-0002-9390-2171

Affiliations

1. National Centre for Epidemiology and Population Health, Australian National University, ACT, Australia
2. School of Medicine and Psychology, Australian National University, ACT, Australia

Additional statistical methods details

We used a grid search to select ARIMA parameters based on information criteria (allowing non-seasonal orders 0-2 and seasonal orders 0-1), informed by assessment of full and partial autocorrelation functions, unit root tests (to determine the required number of differences to achieve stationarity), and residuals diagnostics (1-3). We used transfer functions to model the impacts of two interruptions (the start of the pandemic and introduction of telehealth in April 2020, and the removal of telephone GP CDM services in July 2021). We chose the best fitting transfer functions using Akaike information criteria from the following: pulse (instantaneous change with immediate return to baseline), pulse with decay/growth (instantaneous change with gradual return to baseline), step change (permanent level shift), step change with decay/growth (gradual decay or growth to a permanent level shift), and ramp (linear increase or decrease) (3). We used these models to estimate the counterfactual time series as the fitted time series absent of the modelled interruption impact (1, 2). To account for the potential confounding by public health restrictions in relation to the COVID-19 outbreak, we controlled for COVID-19 cases, modelled using a transfer function that was chosen after examining cross-correlation functions using pre-whitened time series (1, 2).

Supplemental Figures S1—S9 are on pages 2—11.

Supplemental Table S1 is on pages 12—13.

Figure S1: New users of GPMP/TCA services per 1,000 population aged 45-<85 who had no claim for CDM services in the previous two years, by month, January 2018 to December 2022. Panel (a) shows the observed time series (grey points), fitted time series (black line) and estimated counterfactual series in the absence of interruptions (blue solid line), controlling for COVID-19 cases. Panel (b) shows the estimated impact of the interruptions, with 95% confidence intervals.

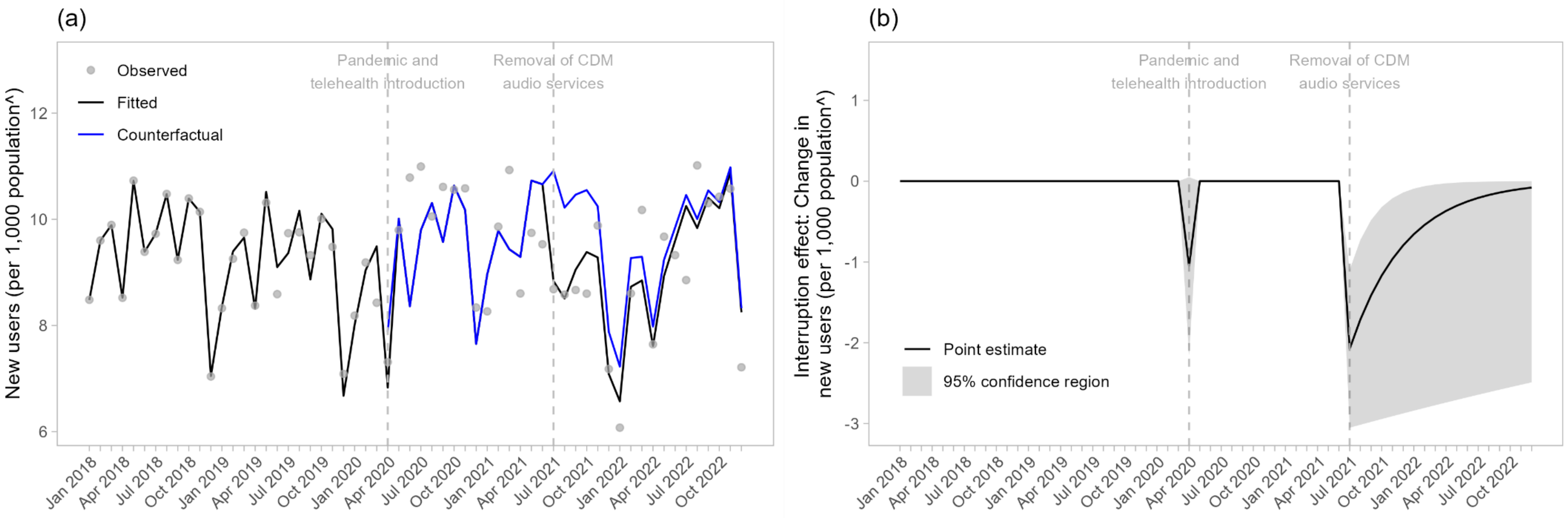

Note. Denominator is the population in each month who were aged 45-<85 years, enrolled in Medicare, alive, residing in one of the eight major states or territories, and not physically absent from Australia. ^Population denominator is restricted to those who had no claim for a GPMP/TCA or review in the previous two years.

Figure S2: CDM uptake, current users per 1,000 population aged 45-<85, by month, January 2018 to December 2022, and by sociodemographic variables: (a) 10-year age group, (b) state or territory, (c) remoteness area, and (d) the Index of Relative Socioeconomic Advantage and Disadvantage (IRSAD).

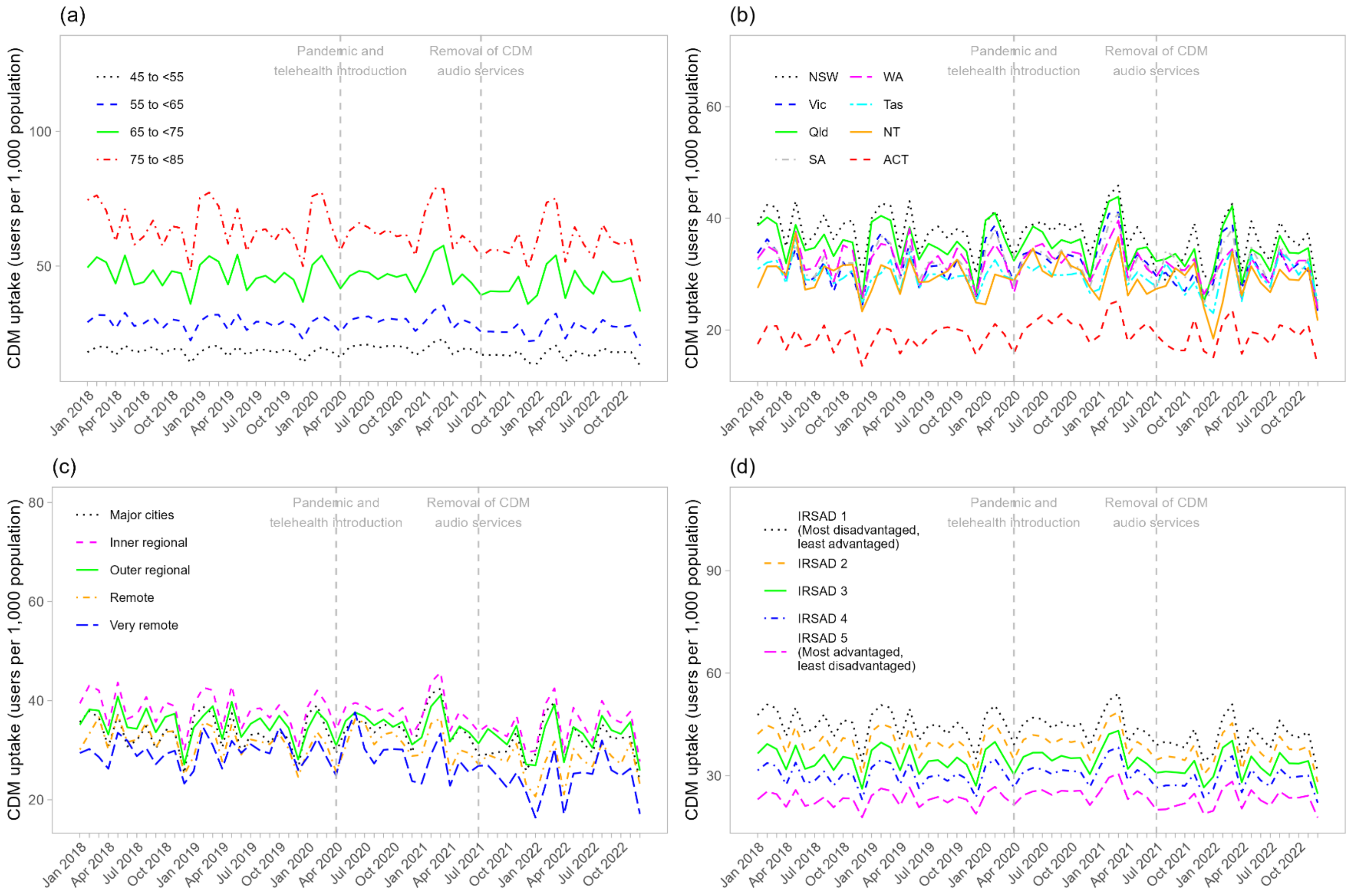

Note. Denominator is the population in each month who were aged 45-<85 years, enrolled in Medicare, alive, residing in one of the eight major states or territories, and not physically absent from Australia.

Figure S3: New users per 1,000 population^ aged 45-<85, by month, January 2018 to December 2022, and by sociodemographic variables: (a) 10-year age group, (b) state or territory, (c) remoteness area, and (d) the Index of Relative Socioeconomic Advantage and Disadvantage (IRSAD).

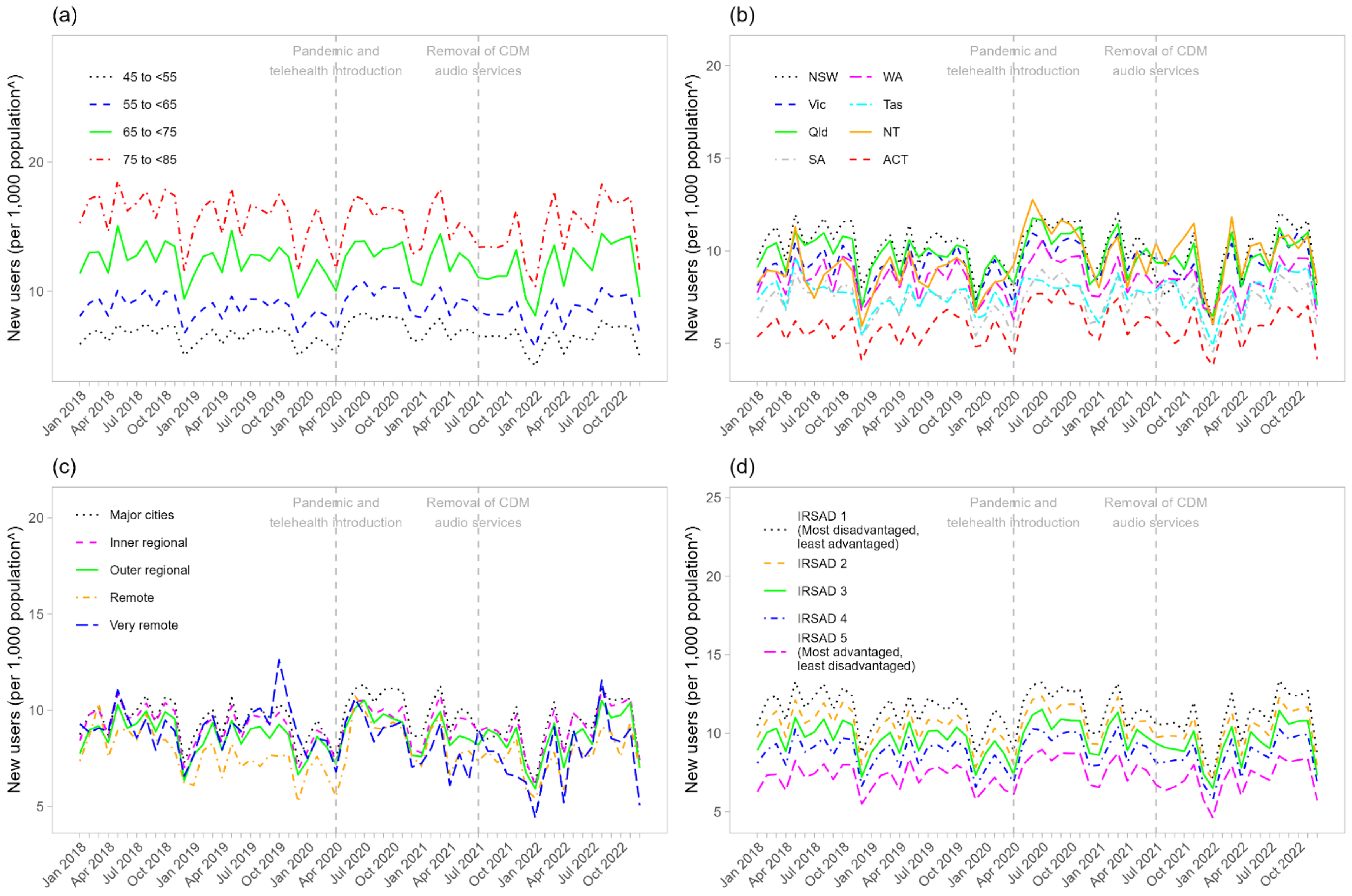

^Note. Denominator is the population in each month who were aged 45-<85 years, enrolled in Medicare, alive, residing in one of the eight major states or territories, not physically absent from Australia, and further restricted to those who had no claim for a GPMP/TCA or review in the previous two years.

Figure S4: Overdue reviews per 1,000 pre-existing users aged 45-<85, by month, January 2018 to December 2022, and by sociodemographic variables: (a) 10-year age group, (b) state or territory, (c) remoteness area, and (d) the Index of Relative Socioeconomic Advantage and Disadvantage (IRSAD).

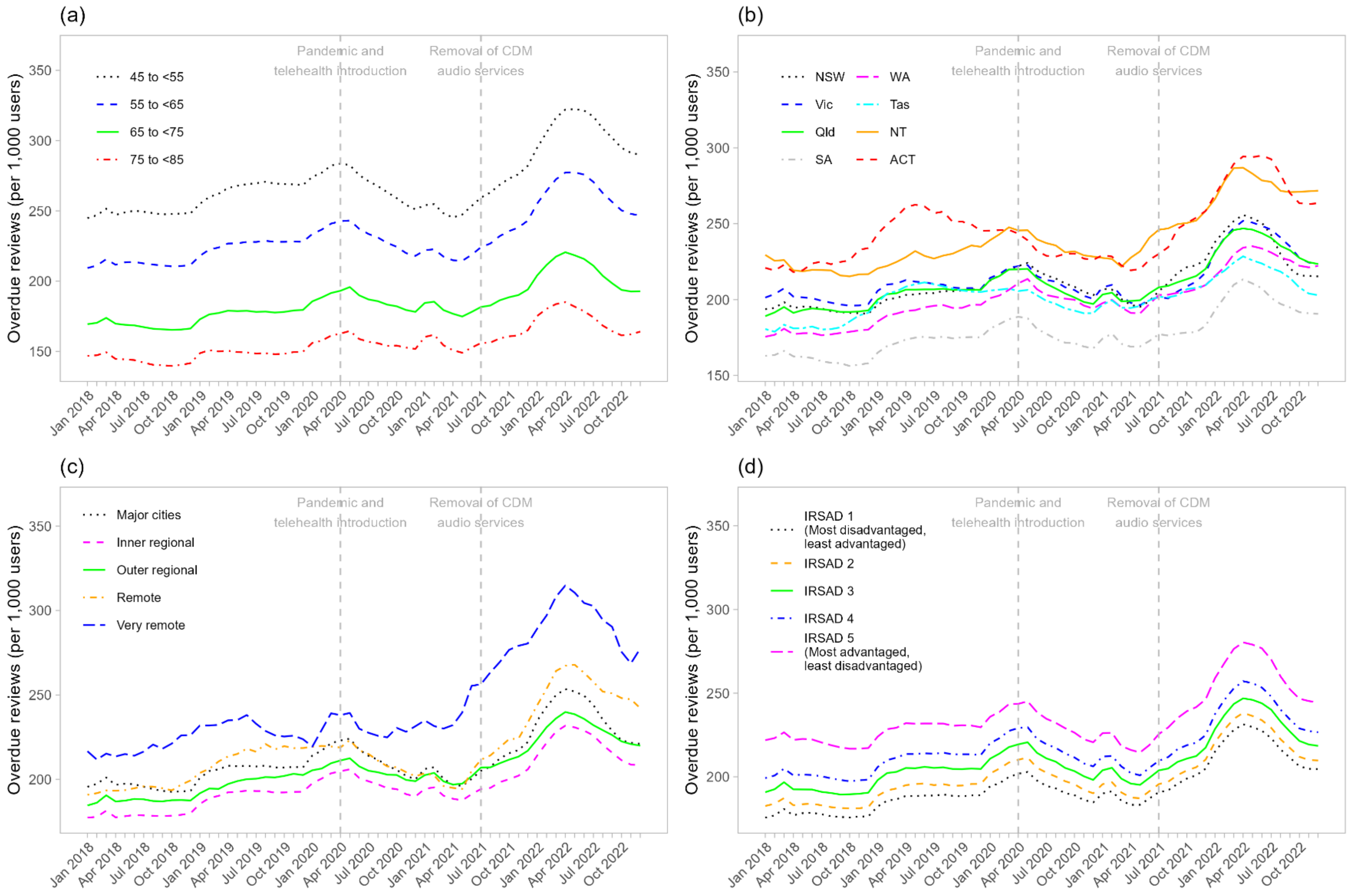

Note. Denominator is the population in each month who were aged 45-<85 years, enrolled in Medicare, alive, residing in one of the eight major states or territories, not physically absent from Australia, and who had a claim for a GPMP/TCA in the previous two years.

Figure S5: Mean days between services, by month, January 2018 to December 2022, and by sociodemographic variables: (a) 10-year age group, (b) state or territory, (c) remoteness area, and (d) the Index of Relative Socioeconomic Advantage and Disadvantage (IRSAD).

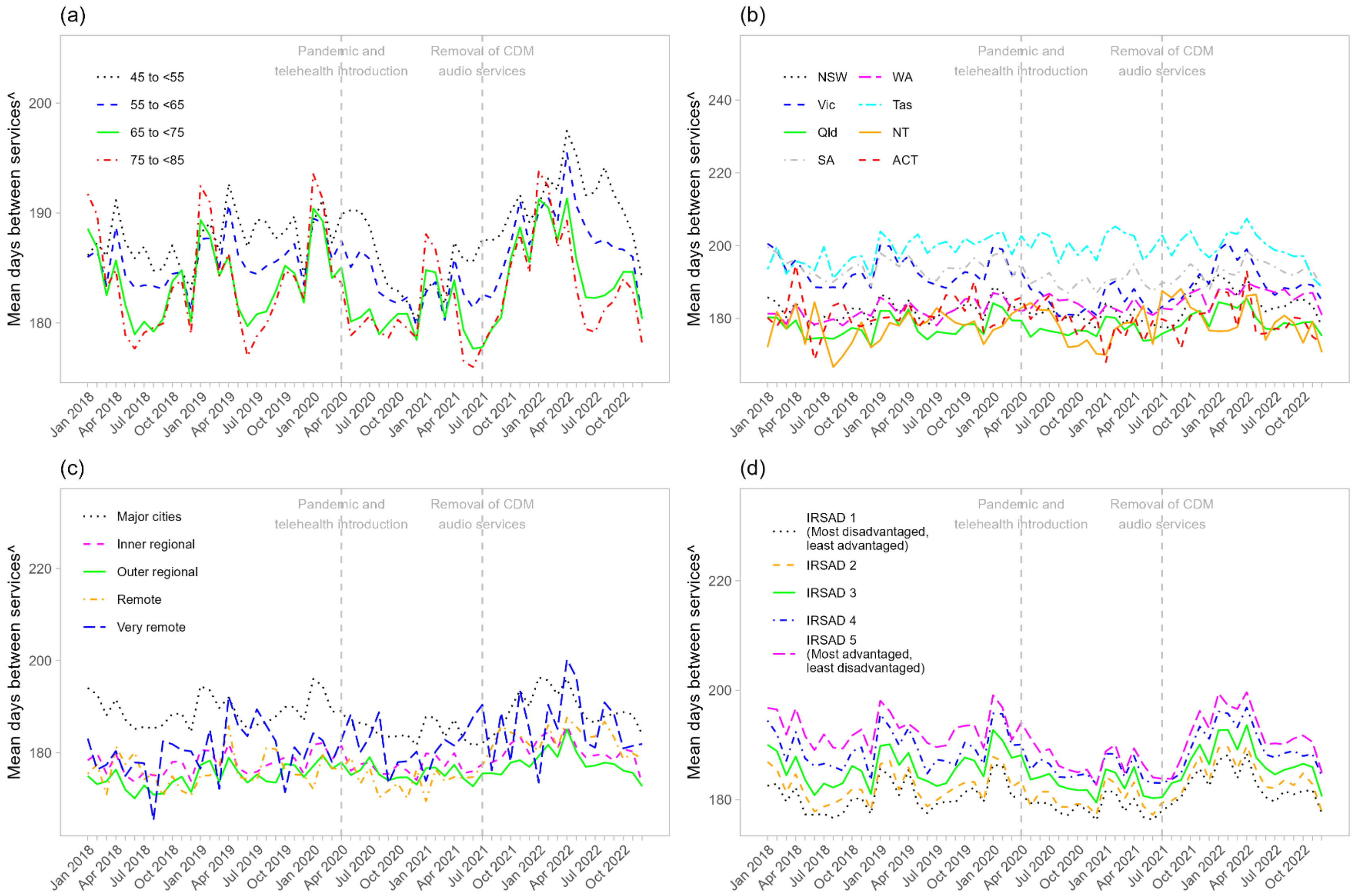

^Note. Denominator is the population in each month who were aged 45-<85 years, enrolled in Medicare, alive, residing in one of the eight major states or territories, not physically absent from Australia, who had a claim for a GPMP/TCA in the previous two years, and further restricted to those who had a review in the current month and at least one previous review since 2016, with time between services capped at 2 years.

Figure S6: CDM monitoring/support services (panels (a) and (b)) and allied health services (panels (c) and (d)), per 1,000 pre-existing users aged 45-<85, by month, January 2018 to December 2022. Panels (a) and (c) show the observed time series (grey points), fitted time series (black line) and estimated counterfactual series in the absence of interruptions (blue solid line), controlling for COVID-19 cases. Panels (b) and (d) show the estimated impact of the interruptions, with 95% confidence intervals.

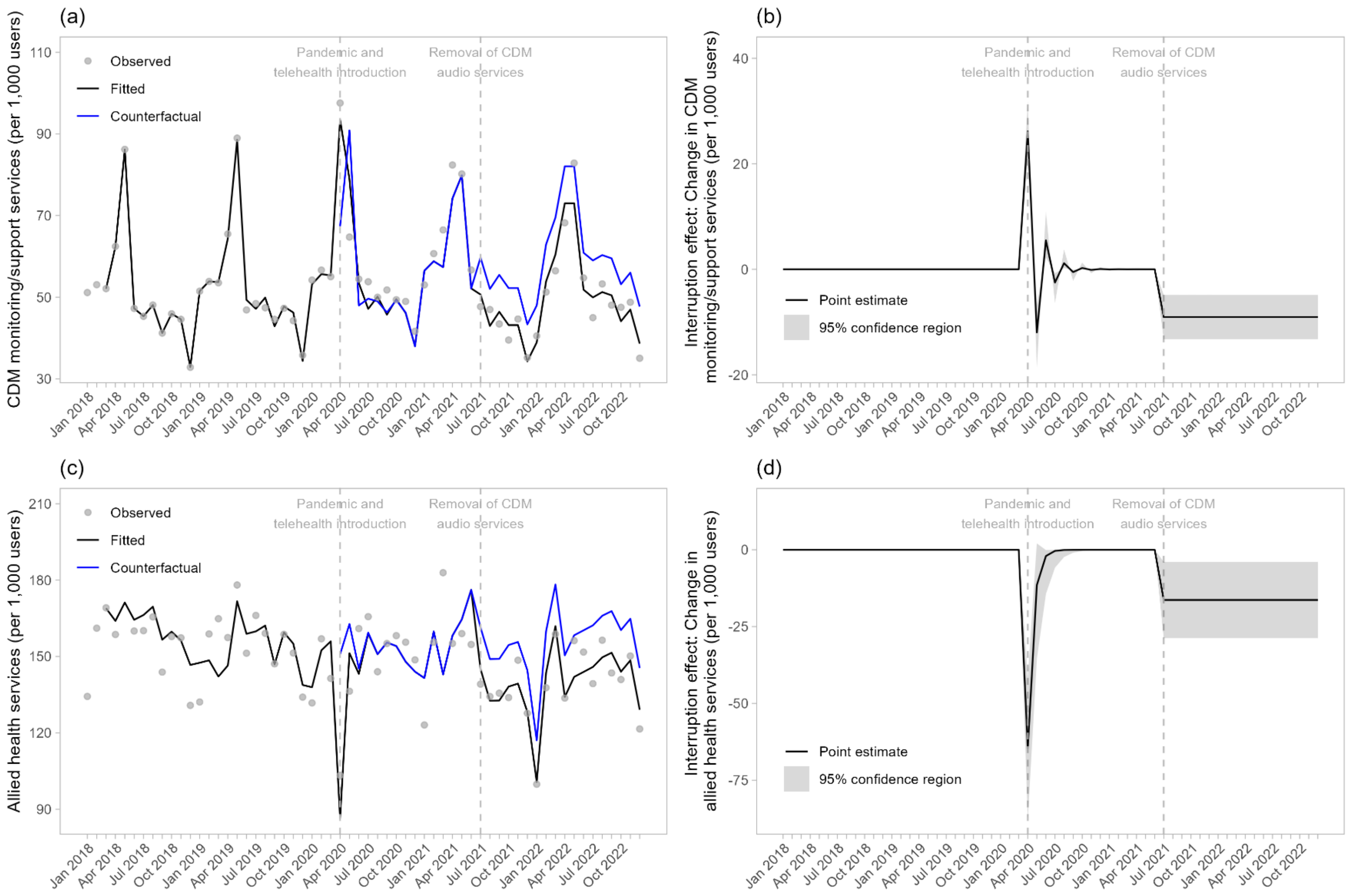

Note. Denominator is the population in each month who were aged 45-<85 years, enrolled in Medicare, alive, residing in one of the eight major states or territories, not physically absent from Australia, and who had a claim for a GPMP/TCA in the previous two years. The oscillation in (b) can be explained as follows: seasonal peaks in CDM monitoring/support services prior to the pandemic occurred every May, but in 2020 the peak occurred in April, a month earlier. The time series model has identified that services would have been lower in April 2020 and then higher in May 2020, were prior seasonal patterns to be repeated.

Figure S7: CDM monitoring/support services (a) and allied health services (b), by month and modality.

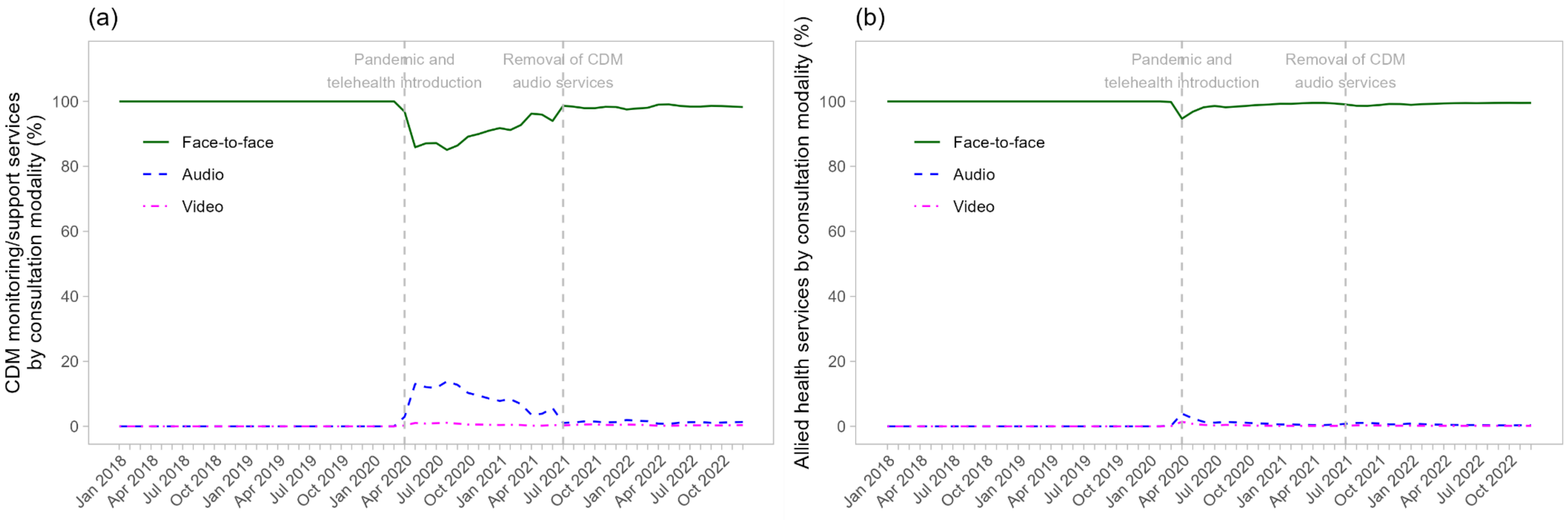

Figure S8: CDM monitoring/support services (a) and allied health services (b) per 1,000 pre-existing users aged 45-<85, by month, January 2018 to December 2022, and by state or territory.

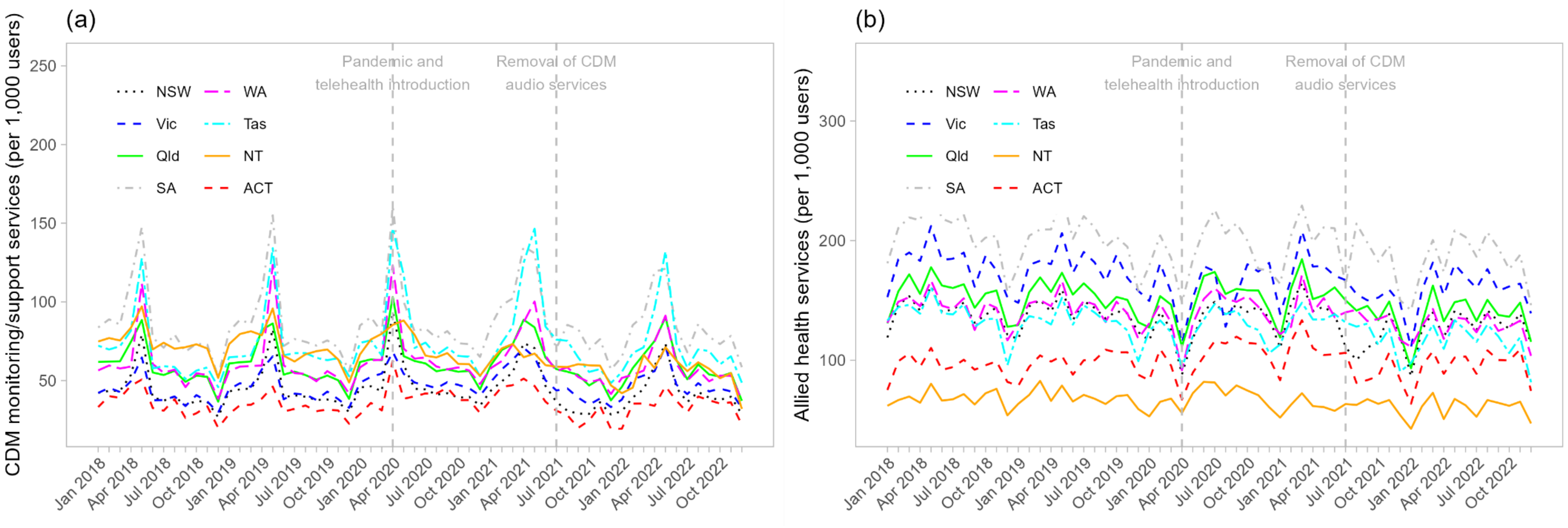

Note. Denominator is the population in each month who were aged 45-<85 years, enrolled in Medicare, alive, residing in one of the eight major states or territories, not physically absent from Australia, and who had a claim for a GPMP/TCA in the previous two years.

Figure S9: Outcome time series for people aged 45<-85 (solid black line) and 45-99 (dashed blue line), by month, January 2018 to December 2022: (a) CDM uptake, current users per 1,000 population; (b) CDM uptake, services per 1,000 population; (c) new users per 1,000 population^; (d) overdue reviews per 1,000 pre-existing users; (e) CDM monitoring/support services per 1,000 pre-existing users; and (f) CDM allied health services per 1,000 pre-existing users.

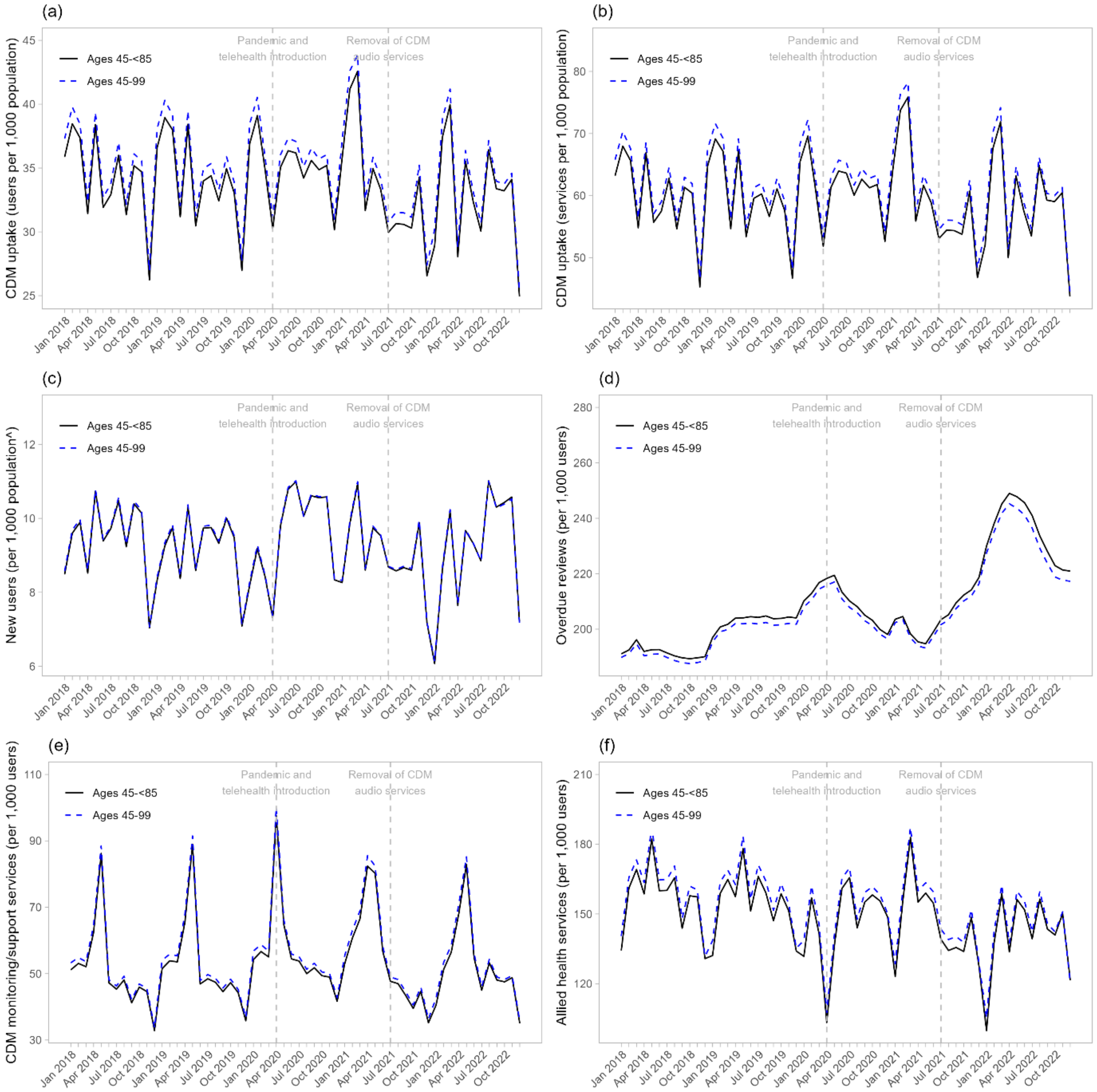

Note: Population denominator refers to the population in each month who were aged 45-<85 years, enrolled in Medicare, alive, residing in one of the eight major states or territories, who were not physically absent from Australia. Users denominator is further restricted to people who had a claim for a GPMP/TCA in the previous two years. ^In panel (c), population denominator is restricted to those who had no claim for a GPMP/TCA or review in the previous two years.

Table S1: Outcome time series, by month, January 2018 to December 2022.

Month	Denominator: population aged 45 to <85†	Denominator: Pre-existing users‡	GPMP/TCA and Review services	CDM monitoring and support services	CDM allied health services	CDM uptake (current users per 1,000 population†)	CDM uptake (services per 1,000 population†)	New users (per 1,000 population^)	Overdue reviews (per 1,000 users‡)	Mean days between services*	CDM monitoring or support services (per 1,000 users‡)	CDM allied health services (per 1,000 users‡)
01/18	9,065,039	2,755,261	573,022	140,908	370,073	35.9	63.2	8.5	191.1	188.5	51.1	134.3
02/18	9,077,059	2,769,852	616,942	146,984	446,263	38.5	68.0	9.6	192.5	187.6	53.1	161.1
03/18	9,046,157	2,778,589	593,995	144,754	469,891	37.4	65.7	9.9	196.2	183.5	52.1	169.1
04/18	9,038,741	2,785,131	495,521	173,914	441,826	31.4	54.8	8.5	192.0	187.1	62.4	158.6
05/18	9,002,134	2,795,317	602,222	240,987	509,270	38.4	66.9	10.7	192.6	182.5	86.2	182.2
06/18	8,995,850	2,812,458	501,153	132,896	450,017	31.9	55.7	9.4	192.6	180.8	47.3	160.0
07/18	9,032,560	2,830,834	519,330	128,301	453,308	32.9	57.5	9.7	191.4	181.8	45.3	160.1
08/18	9,019,258	2,842,856	566,317	136,730	470,626	36.0	62.8	10.5	190.4	181.2	48.1	165.5
09/18	9,029,908	2,863,603	493,468	118,049	411,966	31.3	54.6	9.2	189.7	181.6	41.2	143.9
10/18	9,150,441	2,910,273	561,310	133,707	459,301	35.2	61.3	10.4	189.4	184.3	45.9	157.8
11/18	9,202,070	2,939,892	555,752	131,068	462,535	34.7	60.4	10.1	189.7	184.5	44.6	157.3
12/18	9,109,971	2,929,860	412,760	96,208	383,210	26.2	45.3	7.0	190.1	180.5	32.8	130.8
01/19	9,238,644	2,967,386	598,534	152,769	392,059	36.6	64.8	8.3	197.0	189.6	51.5	132.1
02/19	9,251,695	2,978,721	639,672	160,451	473,165	38.9	69.1	9.3	200.8	188.9	53.9	158.8
03/19	9,234,616	2,976,244	619,926	159,312	490,579	38.0	67.1	9.7	201.8	184.8	53.5	164.8
04/19	9,210,598	2,983,250	503,614	195,379	469,659	31.2	54.7	8.4	204.0	188.3	65.5	157.4
05/19	9,173,407	2,979,530	616,939	265,070	530,575	38.4	67.3	10.3	204.1	183.7	89.0	178.1
06/19	9,177,338	2,986,960	490,148	140,054	451,872	30.5	53.4	8.6	204.5	181.4	46.9	151.3
07/19	9,212,321	2,998,052	548,689	145,255	498,019	34.0	59.6	9.7	204.2	182.5	48.4	166.1
08/19	9,205,390	3,000,315	554,940	142,261	477,313	34.4	60.3	9.8	204.8	183.0	47.4	159.1
09/19	9,205,623	3,016,247	521,425	134,445	443,719	32.4	56.6	9.3	203.8	184.0	44.6	147.1
10/19	9,319,707	3,049,376	569,383	144,213	483,917	34.9	61.1	10.0	203.9	185.8	47.3	158.7
11/19	9,374,440	3,067,453	539,791	135,717	464,263	33.0	57.6	9.5	204.4	185.9	44.2	151.4
12/19	9,281,923	3,055,032	433,580	109,367	409,489	27.0	46.7	7.1	204.1	182.7	35.8	134.0
01/20	9,407,812	3,082,471	616,232	167,073	406,154	36.9	65.5	8.2	210.2	190.9	54.2	131.8
02/20	9,455,418	3,096,749	657,932	175,417	486,022	39.1	69.6	9.2	212.9	190.1	56.6	156.9
03/20	9,576,006	3,123,670	584,087	171,912	441,606	34.9	61.0	8.4	216.8	185.1	55.0	141.4
04/20	9,597,252	3,123,517	497,832	304,732	322,700	30.3	51.9	7.3	218.3	186.0	97.6	103.3
05/20	9,612,969	3,126,784	589,530	202,400	426,341	35.1	61.3	9.8	219.5	182.6	64.7	136.4
06/20	9,630,483	3,148,403	615,958	171,401	506,771	36.4	64.0	10.8	213.4	183.3	54.4	161.0
07/20	9,642,631	3,167,712	613,524	170,384	524,685	36.2	63.6	11.0	210.2	183.4	53.8	165.6
08/20	9,654,767	3,174,728	579,876	158,701	457,203	34.2	60.1	10.1	208.1	181.0	50.0	144.0
09/20	9,664,262	3,195,321	605,496	165,447	495,598	35.6	62.7	10.6	205.1	180.7	51.8	155.1
10/20	9,676,244	3,207,784	592,844	158,409	507,413	34.9	61.3	10.6	203.2	181.2	49.4	158.2

Month	Denominator: population aged 45 to <85†	Denominator: Pre-existing users‡	GPMP/TCA and Review services	CDM monitoring and support services	CDM allied health services	CDM uptake (current users per 1,000 population†)	CDM uptake (services per 1,000 population†)	New users (per 1,000 population^)	Overdue reviews (per 1,000 users‡)	Mean days between services*	CDM monitoring or support services (per 1,000 users‡)	CDM allied health services (per 1,000 users‡)
11/20	9,689,939	3,222,567	598,733	157,782	501,445	35.2	61.8	10.6	199.9	181.0	49.0	155.6
12/20	9,702,294	3,238,968	510,606	134,911	481,554	30.2	52.6	8.3	198.1	179.1	41.7	148.7
01/21	9,714,939	3,241,838	620,945	171,879	399,067	35.8	63.9	8.3	203.6	185.0	53.0	123.1
02/21	9,730,103	3,253,192	717,433	197,406	506,234	41.2	73.7	9.9	204.6	184.8	60.7	155.6
03/21	9,742,036	3,269,554	739,067	217,353	598,027	42.6	75.9	10.9	198.3	181.2	66.5	182.9
04/21	9,750,755	3,277,030	545,033	269,979	508,294	31.7	55.9	8.6	195.4	184.6	82.4	155.1
05/21	9,765,087	3,281,445	602,062	263,152	521,733	34.9	61.7	9.7	194.8	180.4	80.2	159.0
06/21	9,782,870	3,297,165	575,882	186,884	510,105	33.3	58.9	9.5	199.0	179.4	56.7	154.7
07/21	9,801,831	3,297,367	520,378	157,266	458,806	30.0	53.1	8.7	203.4	180.5	47.7	139.1
08/21	9,823,456	3,296,483	534,744	154,885	442,690	30.7	54.4	8.6	205.2	181.3	47.0	134.3
09/21	9,849,281	3,299,505	535,461	143,382	447,363	30.6	54.4	8.7	209.5	182.7	43.5	135.6
10/21	9,871,987	3,296,254	530,905	130,265	441,238	30.3	53.8	8.6	212.3	186.6	39.5	133.9
11/21	9,887,613	3,305,066	600,462	147,598	490,920	34.3	60.7	9.9	214.2	189.5	44.7	148.5
12/21	9,888,646	3,306,629	462,925	116,345	422,265	26.6	46.8	7.2	218.6	186.2	35.2	127.7
01/22	9,914,537	3,292,085	516,258	133,409	328,637	28.9	52.1	6.1	230.0	191.7	40.5	99.8
02/22	9,928,005	3,290,162	670,822	168,653	453,136	37.4	67.6	8.6	238.1	191.7	51.3	137.7
03/22	9,918,051	3,300,480	713,102	186,358	524,086	40.0	71.9	10.2	245.1	188.4	56.5	158.8
04/22	9,899,109	3,293,946	495,085	224,733	440,255	28.1	50.0	7.6	249.0	192.6	68.2	133.7
05/22	9,875,179	3,285,687	623,913	272,258	513,345	35.5	63.2	9.7	247.8	187.5	82.9	156.2
06/22	9,840,478	3,270,921	566,794	179,032	496,293	32.4	57.6	9.3	245.6	184.3	54.7	151.7
07/22	9,845,737	3,251,582	526,803	146,306	453,063	30.1	53.5	8.9	241.0	183.9	45.0	139.3
08/22	9,854,468	3,263,344	639,343	173,803	510,360	36.4	64.9	11.0	233.6	184.9	53.3	156.4
09/22	9,861,263	3,270,095	584,569	157,142	469,325	33.4	59.3	10.3	228.0	184.8	48.1	143.5
10/22	9,916,579	3,283,867	585,485	156,084	462,847	33.2	59.0	10.4	222.9	185.7	47.5	140.9
11/22	9,947,124	3,297,336	601,496	160,828	495,242	34.1	60.5	10.6	221.4	184.9	48.8	150.2
12/22	9,858,120	3,272,700	431,409	114,719	397,762	24.9	43.8	7.2	220.9	180.2	35.1	121.5

Note: †Population denominator refers to the population in each month who were aged 45-<85 years, enrolled in Medicare, alive, residing in one of the eight major states or territories, who were not physically absent from Australia. ‡Pre-existing users denominator is further restricted to people who had a claim for a GPMP/TCA in the previous two years. ^Population denominator is restricted to those who had no claim for a GPMP/TCA or review in the previous two years. *Pre-existing users denominator is further restricted to those who had a review in the current month and at least one previous review since 2016, with time between services capped at 2 years.

References

1. Box GEP, Jenkins GM, Reinsel GC. Time Series Analysis: Wily; 2008.
2. Cryer JD, Chan K-S. Time Series Analysis. With Applications in R: Springer; 2008.
3. Schaffer AL, Dobbins TA, Pearson S-A. Interrupted time series analysis using autoregressive integrated moving average (ARIMA) models: a guide for evaluating large-scale health interventions. BMC Medical Research Methodology. 2021;21(1):58.

The RECORD statement – checklist of items, extended from the STROBE statement, that should be reported in observational studies using routinely collected health data.

	Item No.	STROBE items	Location in manuscript where items are reported	RECORD items	Location in manuscript where items are reported
**Title and abstract**					
	1	(a) Indicate the study's design with a commonly used term in the title or the abstract (b) Provide in the abstract an informative and balanced summary of what was done and what was found	a) Title (p1) Abstract (p2)<br>b) Abstract (p2)	RECORD 1.1: The type of data used should be specified in the title or abstract. When possible, the name of the databases used should be included.<br><br>RECORD 1.2: If applicable, the geographic region and timeframe within which the study took place should be reported in the title or abstract.<br><br>RECORD 1.3: If linkage between databases was conducted for the study, this should be clearly stated in the title or abstract.	Title (p1) Abstract (p2)<br><br>Abstract (p2)<br><br>Abstract (p2)
**Introduction**					
Background rationale	2	Explain the scientific background and rationale for the investigation being reported	Introduction (p4-5)		
Objectives	3	State specific objectives, including any prespecified hypotheses	Introduction (p4-5)		
**Methods**					
Study Design	4	Present key elements of study design early in the paper	Methods (p5-6)		
Setting	5	Describe the setting, locations, and relevant dates, including periods of recruitment, exposure, follow-up, and data collection	Methods (p5-6)		

Participants	6	*(a) Cohort study* - Give the eligibility criteria, and the sources and methods of selection of participants. Describe methods of follow-up<br>*Case-control study* - Give the eligibility criteria, and the sources and methods of case ascertainment and control selection. Give the rationale for the choice of cases and controls<br>*Cross-sectional study* - Give the eligibility criteria, and the sources and methods of selection of participants<br><br>*(b) Cohort study* - For matched studies, give matching criteria and number of exposed and unexposed<br>*Case-control study* - For matched studies, give matching criteria and the number of controls per case	a) Methods (p6)<br><br>b) N/A	RECORD 6.1: The methods of study population selection (such as codes or algorithms used to identify subjects) should be listed in detail. If this is not possible, an explanation should be provided.<br><br>RECORD 6.2: Any validation studies of the codes or algorithms used to select the population should be referenced. If validation was conducted for this study and not published elsewhere, detailed methods and results should be provided.<br><br>RECORD 6.3: If the study involved linkage of databases, consider use of a flow diagram or other graphical display to demonstrate the data linkage process, including the number of individuals with linked data at each stage.	Methods (p6-7),<br><br>N/A<br><br>N/A
Variables	7	Clearly define all outcomes, exposures, predictors, potential confounders, and effect modifiers. Give diagnostic criteria, if applicable.	Methods (p6-8)	RECORD 7.1: A complete list of codes and algorithms used to classify exposures, outcomes, confounders, and effect modifiers should be provided. If these cannot be reported, an explanation should be provided.	Methods (p6-8),
Data sources/ measurement	8	For each variable of interest, give sources of data and details of methods of assessment (measurement).<br>Describe comparability of assessment methods if there is more than one group	Methods (p6-8)		

Bias	9	Describe any efforts to address potential sources of bias			
Study size	10	Explain how the study size was arrived at	Methods (p6)		
Quantitative variables	11	Explain how quantitative variables were handled in the analyses. If applicable, describe which groupings were chosen, and why	Methods (p8)		
Statistical methods	12	(a) Describe all statistical methods, including those used to control for confounding<br>(b) Describe any methods used to examine subgroups and interactions<br>(c) Explain how missing data were addressed<br>(d) *Cohort study* - If applicable, explain how loss to follow-up was addressed<br>*Case-control study* - If applicable, explain how matching of cases and controls was addressed<br>*Cross-sectional study* - If applicable, describe analytical methods taking account of sampling strategy<br>(e) Describe any sensitivity analyses	a) Methods (p8)<br>b) Methods (p8)<br>c) N/A (no missing data MBS)<br>d) N/A, whole of population data were used<br>e) Methods (p8)		
Data access and cleaning methods		..		RECORD 12.1: Authors should describe the extent to which the investigators had access to the database population used to create the study population.	Data statement (p19)

				RECORD 12.2: Authors should provide information on the data cleaning methods used in the study.	N/A
Linkage		..		RECORD 12.3: State whether the study included person-level, institutional-level, or other data linkage across two or more databases. The methods of linkage and methods of linkage quality evaluation should be provided.	Methods (p5-6)
**Results**					
Participants	13	(a) Report the numbers of individuals at each stage of the study (*e.g.*, numbers potentially eligible, examined for eligibility, confirmed eligible, included in the study, completing follow-up, and analysed)<br>(b) Give reasons for non-participation at each stage.<br>(c) Consider use of a flow diagram	Supplementary table S1	RECORD 13.1: Describe in detail the selection of the persons included in the study (*i.e.,* study population selection) including filtering based on data quality, data availability and linkage. The selection of included persons can be described in the text and/or by means of the study flow diagram.	Results (p9)
Descriptive data	14	(a) Give characteristics of study participants (*e.g.*, demographic, clinical, social) and information on exposures and potential confounders<br>(b) Indicate the number of participants with missing data for each variable of interest<br>(c) *Cohort study* - summarise follow-up time (*e.g.*, average and total amount)	a) Supplementary table S1<br>b) N/A<br>c) N/A		
Outcome data	15	*Cohort study* - Report numbers of outcome events or summary measures over time			

		Case-control study - Report numbers in each exposure category, or summary measures of exposure<br>*Cross-sectional study* - Report numbers of outcome events or summary measures	Figure 1-4 (p12-15)		
Main results	16	(a) Give unadjusted estimates and, if applicable, confounder-adjusted estimates and their precision (e.g., 95% confidence interval). Make clear which confounders were adjusted for and why they were included<br>(b) Report category boundaries when continuous variables were categorized<br>(c) If relevant, consider translating estimates of relative risk into absolute risk for a meaningful time period	a) Supplementary table S1<br>b) N/A (no continuous variables)<br>c) N/A		
Other analyses	17	Report other analyses done—e.g., analyses of subgroups and interactions, and sensitivity analyses	Supplementary figures S1-13, supplementary table S1		
**Discussion**					
Key results	18	Summarise key results with reference to study objectives	Discussion (p16)		
Limitations	19	Discuss limitations of the study, taking into account sources of potential bias or imprecision. Discuss both direction and magnitude of any potential bias	Discussion (p17)	RECORD 19.1: Discuss the implications of using data that were not created or collected to answer the specific research question(s). Include discussion of misclassification bias, unmeasured confounding, missing data, and changing	Discussion (p17)

				eligibility over time, as they pertain to the study being reported.	
Interpretation	20	Give a cautious overall interpretation of results considering objectives, limitations, multiplicity of analyses, results from similar studies, and other relevant evidence	Discussion (p18-19)		
Generalisability	21	Discuss the generalisability (external validity) of the study results	Discussion (p16-18)		
**Other Information**					
Funding	22	Give the source of funding and the role of the funders for the present study and, if applicable, for the original study on which the present article is based	Funding (p19)		
Accessibility of protocol, raw data, and programming code		..		RECORD 22.1: Authors should provide information on how to access any supplemental information such as the study protocol, raw data, or programming code.	Data statement (p19)

*Reference: Benchimol EI, Smeeth L, Guttmann A, Harron K, Moher D, Petersen I, Sørensen HT, von Elm E, Langan SM, the RECORD Working Committee. The REporting of studies Conducted using Observational Routinely-collected health Data (RECORD) Statement. *PLoS Medicine* 2015; in press.

*Checklist is protected under Creative Commons Attribution (CC BY) license.
